# Nonlinear relationship between fibrinogen-to-albumin ratio and mortality in critically ill patients with sepsis: a retrospective cohort study

**DOI:** 10.3389/fnut.2025.1562091

**Published:** 2025-06-18

**Authors:** Daishan Jiang, Xiaoyu Yuan, Yanbo Shen, Tingting Bian

**Affiliations:** ^1^Department of Emergency Medicine, Affiliated Hospital of Nantong University, Nantong, Jiangsu, China; ^2^Department of Pathology, Affiliated Hospital of Nantong University, Nantong, Jiangsu, China

**Keywords:** sepsis, fibrinogen-to-albumin ratio, mortality, biomarkers, MIMIC database

## Abstract

**Background:**

The fibrinogen-albumin ratio (FAR) is recognized as a prognostic biomarker in several diseases, but its role in sepsis remains controversial. To elucidate the relationship between FAR and mortality risk in a large cohort of patients with sepsis.

**Methods:**

In this retrospective cohort study, we analyzed clinical data from the Medical Information Mart for Intensive Care IV Database (version 2.2) to investigate the mortality of sepsis patients. We employed restricted cubic spline curves and Cox regression models to evaluate the effect of FAR on mortality and conducted subgroup analyses to verify the consistency of our primary findings.

**Results:**

In our analysis of 4,615 sepsis patients, we observed that mortality risk initially decreased with increasing FAR values, reaching a minimum at approximately 94.5*10^−3^, before rising again. Cox regression analysis revealed differing hazard ratios (HRs) for FAR quartiles relative to the second quartile (Q2). At 28 days, adjusted HRs were 1.23 (95% CI: 1.03–1.46) for Q1, 1.14 (0.96–1.36) for Q3, and 1.11 (0.93–1.33) for Q4. By 90 days, these HRs adjusted to 1.25 (1.07–1.46) for Q1, 1.21 (1.04–1.41) for Q3, and 1.21 (1.03–1.42) for Q4. This pattern persisted at 1-year mortality, with HRs of 1.16 (1.00–1.33) for Q1, 1.22 (1.06–1.39) for Q3, and 1.24 (1.07–1.43) for Q4.

**Conclusion:**

FAR exhibited a nonlinear, U-shaped association with mortality risk at 28 days, 90 days, and 1 year in patients with sepsis. These findings suggest that FAR may serve as a practical prognostic biomarker to support early risk stratification and clinical decision-making in sepsis care.

## Introduction

Sepsis, defined as a severe illness caused by a dysregulated response to infection leading to acute organ dysfunction, presents significant challenges in healthcare. This condition is characterized by a range of physiological, biological, and biochemical abnormalities resulting from an inappropriate reaction to infection, which can lead to multiple organ dysfunction syndrome and death (1). The most recent sepsis management guidelines underscore the significance of prompt detection while advising caution against exclusive reliance on the quick sequential organ failure assessment (qSOFA) due to its restricted sensitivity (2). The complexity of sepsis presents a significant challenge for emergency physicians tasked with making prognostic assessments for sepsis patients and has made the investigation of potentially prognostic prediction tools for sepsis a significant area of research. The emergency department (ED) plays a critical role in the early diagnosis, severity assessment, and initiation of intensive treatment for sepsis patients. However, the constrained resources in the emergency setting present challenges to emergency physicians. Our prior research identified certain combinations of commonly utilized markers that may be associated with sepsis outcomes (3).

Fibrinogen, a critical plasma protein, is essential for hemostasis, wound healing, and immune defense (4). It facilitates platelet aggregation, serves as a scaffold for tissue regeneration, and modulates immune responses (5, 6). Elevated fibrinogen levels are associated with atherosclerotic cardiovascular disease, functioning as both a potential etiological factor and a biomarker (7). Albumin, the predominant plasma protein, is crucial for regulating blood pressure, facilitating molecular transport, and exhibiting antioxidant and anti-inflammatory properties (8). Additionally, it serves as a prognostic biomarker for disease progression and mortality risk, with decreased levels often indicating unfavorable clinical outcomes across various medical contexts (8–10). The fibrinogen-to-albumin ratio (FAR) is posited to simultaneously reflect nutritional and inflammatory statuses, potentially offering enhanced prognostic precision beyond what each biomarker could independently. Fibrinogen and albumin tests are bound to coagulation and liver function tests, respectively, and are routinely examined in Chinese hospitals for patients with sepsis in the ED. Elevated FAR levels have been linked to a heightened risk of adverse outcomes in coronary heart disease, stroke, chronic kidney disease, COVID-19, and cancer (11–17). In sepsis studies, FAR was initially identified as an independent predictor of short-term prognosis in patients undergoing surgery for sepsis due to peritonitis (18). Similar findings have been reported in studies of sepsis caused by various infections and neonatal sepsis (19, 20). Conversely, another study found that lower FAR was associated with higher 30-day mortality, although these results did not reach statistical significance in adjusted analyses (21). Thus, the role of FAR in predicting sepsis outcomes remains complex and uncertain.

Our study examined the association between FAR and mortality among critically ill adult sepsis patients by analyzing data from the extensive patient cohort in the Medical Information Mart for Intensive Care (MIMIC) IV database through Cox regression analysis and restricted cubic spline. We sought to expand the repertoire of early disease severity identification strategies in sepsis patients by assessing FAR’s prognostic impact in a substantial sepsis patient cohort.

## Methods

### Data source and study design

The study extracted data from the MIMIC-IV database (version 2.2), a successor to MIMIC-III, which received institutional review board approval (22). This investigation included patients admitted to intensive care units (ICUs) at Beth Israel Deaconess Medical Center (BIDMC) between 2008 and 2019. The MIMIC-IV database, developed by the Computational Physiology Laboratory of Massachusetts Institute of Technology, encompasses desensitization data for over 50,000 critically ill patients at BIDMC during this period. It provides comprehensive information, including demographics, laboratory indicators, vital signs, and medications. To access the database, the author (DJ) completed a Protecting Human Research Participants course, receiving certification (number: 31591048). This retrospective cohort study explored the relationship between the FAR and mortality risk in critically ill patients with sepsis.

### Study population selection

**Figure 1:**
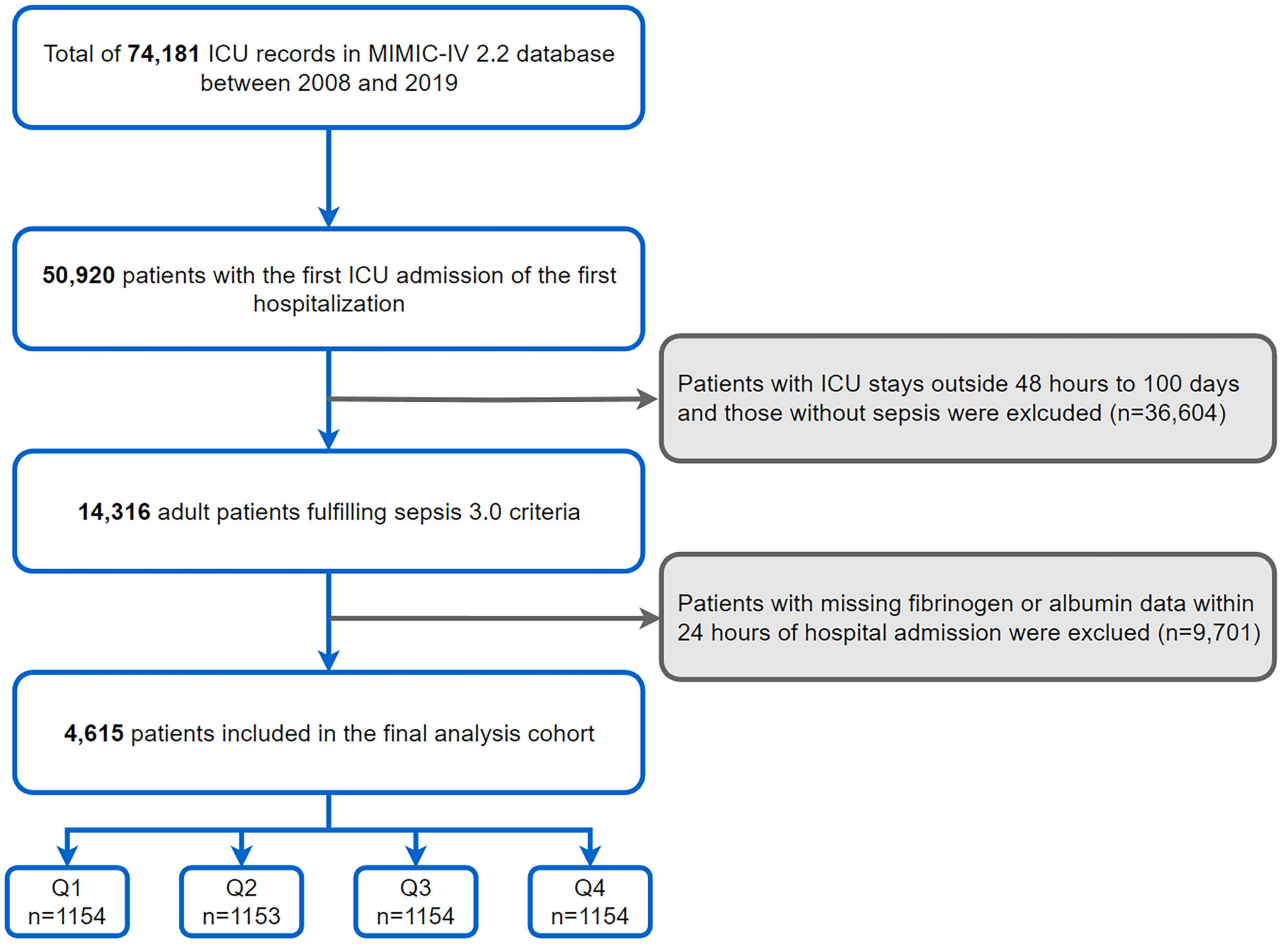
Flow chart showing patient screening.

This investigation was conducted among 50,920 adults with the first ICU admission of the first hospitalization as recorded in the MIMIC-IV database. Inclusion was specific to those diagnosed with sepsis, adhering to the Sepsis-3 criteria from the Third International Consensus Definitions for Sepsis (1). Exclusion criteria encompassed: (1) ICU stays <48 h or more than 100 days; (2) unrecorded fibrinogen or albumin within 24 h of admission (Figure 1).

### Data extraction

We extracted patient data from the MIMIC-IV database within the first 24 h post-admission using Structured Query Language, focusing on the following variables: (1) Comorbidities, including myocardial infarction, congestive heart failure, peripheral vascular disease, cerebrovascular disease, diabetes mellitus, chronic pulmonary disease, severe liver disease, chronic kidney disease, malignant cancer, and metastatic solid tumor; (2) Vital signs, including heart rate, respiratory rate, mean blood pressure, temperature, and percutaneous oxygen saturation (SPO_2_) (3) Laboratory parameters, covering white blood cell count, hemoglobin, platelet count, total bilirubin, albumin, anion gap, bicarbonate, blood urea nitrogen (BUN), creatinine, glucose, calcium, chloride, sodium, potassium, and fibrinogen; (4) Scoring systems such as the acute physiology score III (APS III) and sequential organ failure assessment (SOFA) score; and (5) Organ function support measures, including the use of vasopressors use, inotropic use, invasive ventilation, and continuous renal replacement therapy (CRRT).

### Definition and endpoints

The study specifies FAR as fibrinogen (g/L) divided by albumin (g/L). The primary outcome measured is mortality risk, evaluated at 28 days, 90 days, and 1 year. Survival data for these intervals were obtained from the MIMIC-IV database.

### Statistical analysis

The present study stratified baseline characteristics of patients into FAR quartiles. Categorical variables were presented as frequencies or percentages, with the chi-square test employed for their comparative analysis. Continuous variables were summarized using medians and interquartile ranges, utilizing nonparametric methods for robust comparisons across distributions. The Kruskal-Wallis H test evaluated differences in continuous variables.

We employed restricted cubic splines (RCS) with five knots at the 5th, 35th, 50th, 65th, and 95th percentiles to explore the FAR-mortality relationship in septic critically ill patients. We estimated survival status using the Kaplan–Meier survival curve, comparing curve differences through the Log-rank test. Cox proportional hazard models assessed the association between FAR and 28-day, 90-day, and 1-year mortality, presenting results as hazard ratios (HR) with 95% confidence intervals (CIs). We employed two models based on FAR quartiles to enhance analytical robustness: unadjusted (Model 1) and adjusted (Model 2). Model 2 used backward stepwise covariate selection, adjusting for age, gender, comorbidities, vital signs, and organ function support measures. All covariates’ variance inflation factor (VIF) remained below 10 (Supplementary Table S1). Missing data, <5% for all variables, were imputed using multiple imputation methods, with further details in the Supplementary Table S2.

Further subgroup analyses were performed based on gender, age (<70 and ≥70 years), comorbidities (chronic kidney disease, severe liver disease, and metastatic solid tumors), and severity of sepsis (SOFA score <4 and ≥4) to assess the consistency of the predictive value of the FAR for hazard ratios of 28-day, 90-day and 1-year mortality. The interactions between FAR and variables used for stratification were examined with likelihood ratio tests. All analyses utilized R statistical software (The R Foundation)^1^ and Free Statistics software (version 1.9). Statistical significance was determined at a *p* < 0.05, with all tests being two-tailed. The report aligns with the STROBE (Strengthening the Reporting of Observational Studies in Epidemiology) guidelines (23).

## Results

### Baseline characteristics

This study included 4,615 critically ill sepsis patients, comprising 2,751 men (59.6%) and 1,864 women, based on specific inclusion and exclusion criteria. These patients were categorized into four groups according to FAR (*10^−3^) quartiles (First quartile (Q1): ≤60.4, Second quartile (Q2): 60.4–94.6, Third quartile (Q3): 94.6–160.6, Forth quartile (Q4): ≥160.6). Patients with higher FAR appear to have a greater age, faster heart and respiratory rates, higher body temperatures, and lower SPO_2_, as well as higher levels of leukocytes, hemoglobin, platelets, BUN, creatinine, blood glucose, fibrinogen, and lower levels of albumin, ALT, AST, total bilirubin, calcium, chloride, sodium, and fewer inotropic drugs use (Table 1).

**Table 1:** Baseline characteristics.

Variables	Fibrinogen to albumin ratio group				*P*-value
	First quartile (*n* = 1,154)	Second quartile (*n* = 1,153)	Third quartile (*n* = 1,154)	Forth quartile (*n* = 1,154)	
FAR, *10^−3^	< 60.4	60.4–94.6	94.6–160.6	> 160.6	< 0.001
Age, years	60 (50, 71)	63 (51, 75)	66 (54, 77)	66 (54, 77)	< 0.001
Gender, *n* (%)					0.19
Female	460 (39.9)	443 (38.4)	463 (40.1)	498 (43.2)	
Male	694 (60.1)	710 (61.6)	691 (59.9)	656 (56.8)	
Vital sign					
Heat rate, beats/min	89 (78, 103)	88 (75, 103)	92 (78, 108)	98 (83, 115)	< 0.001
Mean blood pressure, mmHg	79 (68, 93)	81 (69, 94)	80 (68, 93)	79 (68, 91)	0.104
Respiratory rate, breaths/min	18 (15, 22)	18 (16, 23)	20 (16, 24)	21 (17, 26)	< 0.001
Temperature, °C	36.6 (36.3, 37.0)	36.7 (36.3, 37.1)	36.8 (36.4, 37.2)	36.8 (36.4, 37.3)	< 0.001
SPO_2_, %	99 (96, 100)	99 (96, 100)	98 (95, 100)	97 (94, 100)	< 0.001
Comorbidities, *n* (%)					
Myocardial infarction	155 (13.4)	233 (20.2)	234 (20.3)	214 (18.5)	< 0.001
Congestive heart failure	261 (22.6)	339 (29.4)	392 (34)	367 (31.8)	< 0.001
Diabetes mellitus	243 (21.1)	294 (25.5)	370 (32.1)	358 (31)	< 0.001
Peripheral vascular disease	192 (16.6)	169 (14.7)	141 (12.2)	132 (11.4)	< 0.001
Cerebrovascular disease	149 (12.9)	183 (15.9)	199 (17.2)	177 (15.3)	0.033
Chronic pulmonary disease	239 (20.7)	284 (24.6)	297 (25.7)	299 (25.9)	0.011
Chronic kidney disease	188 (16.3)	216 (18.7)	260 (22.5)	272 (23.6)	< 0.001
Severe liver disease	362 (31.4)	183 (15.9)	106 (9.2)	36 (3.1)	< 0.001
Malignant cancer	117 (10.1)	135 (11.7)	185 (16)	205 (17.8)	< 0.001
Scoring					
APS III	57.0 (41.0, 75.8)	53.0 (39.0, 70.0)	57.0 (43.0, 72.0)	62.0 (49.0, 79.0)	< 0.001
SOFA	9.0 (6.0, 12.0)	8.0 (5.0, 10.0)	7.0 (5.0, 10.0)	8.0 (5.0, 11.0)	< 0.001
Laboratory					
White blood cell, 10^9^/L	11.1 (7.5, 16.1)	11.6 (7.9, 16.7)	12.2 (8.2, 17.7)	12.8 (8.2, 19.1)	< 0.001
Hemoglobin, g/dL	10.5 (8.3, 12.7)	11.1 (9.1, 13.0)	10.9 (9.1, 12.7)	10.5 (8.9, 12.4)	< 0.001
Platelets, 10^9^/L	133.5 (76.0, 202.8)	173.0 (117.0, 241.0)	182.0 (122.0, 258.2)	197.5 (123.2, 292.8)	< 0.001
Total bilirubin, mg/dL	1.4 (0.6, 4.3)	0.9 (0.5, 2.0)	0.7 (0.4, 1.5)	0.7 (0.4, 1.4)	< 0.001
BUN, mg/dL	20 (14, 34)	20 (15, 33)	25 (16, 42)	30 (18, 51)	< 0.001
Creatinine, mg/dL	1.1 (0.8, 1.7)	1.1 (0.8, 1.6)	1.2 (0.9, 2.1)	1.3 (0.9, 2.4)	< 0.001
Glucose, mg/dL	132 (107, 173)	136 (107, 179)	138 (112, 184)	136 (108, 181)	0.021
Calcium, mg/dL	8.3 (7.7, 9.0)	8.2 (7.6, 8.8)	8.1 (7.5, 8.7)	8.0 (7.3, 8.5)	< 0.001
Chloride, mmol/L	105 (99, 109)	104 (100, 108)	103 (99, 108)	103 (98, 108)	< 0.001
Sodium, mmol/L	139 (136, 142)	139 (136, 141)	138 (135, 141)	138 (134, 141)	< 0.001
Potassium, mmol/L	4.2 (3.7, 4.8)	4.3 (3.8, 4.8)	4.2 (3.7, 4.8)	4.2 (3.7, 4.8)	0.311
Fibrinogen, g/L	1.4 (1.1, 1.7)	2.37 (1.9, 2.7)	3.61 (2.9, 4.3)	6.1 (5.0, 7.4)	< 0.001
Albumin, g/L	33 (28, 38)	31 (27, 36)	30 (25, 34)	25 (22, 29)	< 0.001
Curing, *n* (%)					
Vasopressor, *n* (%)	627 (54.3)	595 (51.6)	688 (59.6)	790 (68.5)	< 0.001
CRRT, *n* (%)	231 (20)	157 (13.6)	175 (15.2)	190 (16.5)	< 0.001
Invasive ventilation, *n* (%)	951 (82.4)	937 (81.3)	910 (78.9)	913 (79.1)	0.091
Mortality, *n* (%)					
In-hospital	314 (27.2)	248 (21.5)	299 (25.9)	336 (29.1)	< 0.001
28-day	333 (28.9)	264 (22.9)	337 (29.2)	344 (29.8)	< 0.001
90-day	404 (35)	325 (28.2)	431 (37.3)	452 (39.2)	< 0.001
1-year	458 (39.7)	410 (35.6)	526 (45.6)	560 (48.5)	< 0.001

FAR, fibrinogen to albumin ratio; SPO_2_, percutaneous oxygen saturation; SOFA, sequential organ failure assessment; APS III, acute physiology score III; BUN, blood urea nitrogen; CRRT, continuous renal replacement therapy.

Regarding comorbidities, higher FAR was associated with increased incidences of myocardial infarction, congestive heart failure, diabetes mellitus, cerebrovascular disease, chronic pulmonary disease, chronic kidney disease, malignant cancer, and metastatic solid tumor, but lower incidences of peripheral vascular disease and severe liver disease. No significant differences were observed in gender distribution, mean blood pressure, anion gap, bicarbonate, potassium, or invasive mechanical ventilation across the groups.

### Association between FAR and sepsis prognosis

In the cohort, the 28-day mortality rate was 27.7% (1,278 patients), the 90-day mortality rate was 34.9% (1,612 patients), and the 1-year mortality rate was 42.3% (1,954 patients). Significantly, the second quartile group exhibited lower SOFA and APSIII scores and decreased usage of vasopressors and CRRT compared to other groups. Concurrently, this group showed the lowest rates of in-hospital mortality and 28-day, 90-day, and 1-year mortality.

**Figure 2:**
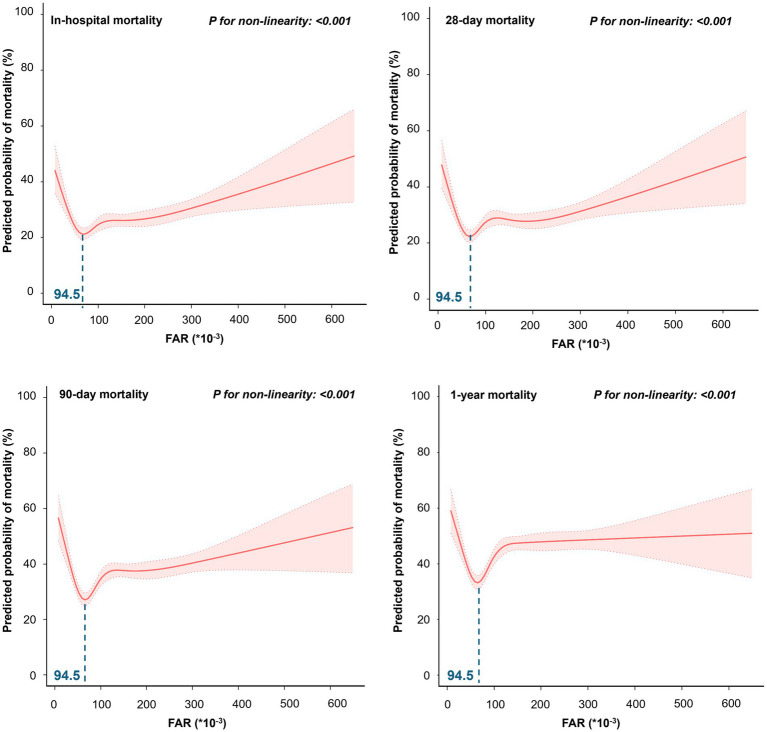
The restricted cubic spline for the association between FAR and predicted probability of mortality.

**Figure 3:**
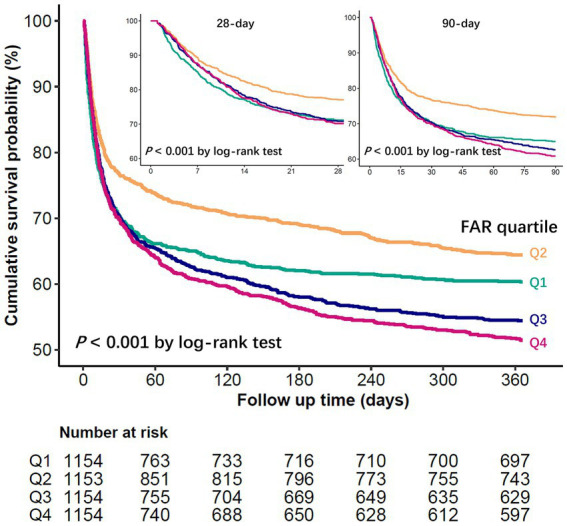
Kaplan–Meier survival curve for mortality according to FAR.

Restricted cubic spline regression models showed that the probability of in-hospital, 28-day, 90-day, and 1-year mortality in patients with sepsis first decreased with increasing FAR values, with the lowest risk at a FAR value of approximately 94.5 (*10^−3^) and then began to increase (*P* for non-linearity < 0.001) (Figure 2). The Kaplan–Meier curve demonstrates the survival rates for the four FAR quartiles. Notably, cumulative survival rates in the Q2 group surpassed those in the Q1, Q3, and Q4 groups at 28 days, 90 days, and 1 year (*p* < 0.001 by log-rank test) (Figure 3).

### FAR and 28-day mortality risk

Using the Cox regression model, we evaluated the hazard ratios (HRs) for all-cause mortality risks with FAR (Table 2). Group Q2, exhibiting the lowest mortality rate, served as the reference. Groups Q1, Q3, and Q4, representing lower and higher FARs, respectively, demonstrated elevated 28-day mortality risks with unadjusted HRs of 1.33 (1.13–1.56), 1.32 (1.12–1.54), and 1.34 (1.14–1.58). Post-adjustment, only the heightened mortality risk in group Q1 remained significant, with adjusted HR of 1.23 (1.03–1.46), while the risks for groups Q3 and Q4 were not significantly altered, with adjusted HRs of 1.14 (0.96–1.36) and 1.11 (0.93–1.33).

**Table 2:** Hazard ratios of all-cause mortality according to FAR.

Mortality	FAR	Events, *n* (%)	Crude HR (95%CI)	*P*-value	Adjusted HR (95%CI)	*P*-value
28-day	Q1	333 (28.9)	1.33 (1.13–1.56)	0.001	1.23 (1.03–1.46)	0.02
	Q2	264 (22.9)	1	Ref.	1	Ref.
	Q3	337 (29.2)	1.32 (1.12–1.54)	0.001	1.14 (0.96–1.36)	0.129
	Q4	344 (29.8)	1.34 (1.14–1.58)	<0.001	1.11 (0.93–1.33)	0.246
90-day	Q1	404 (35)	1.32 (1.14–1.53)	<0.001	1.25 (1.07–1.46)	0.005
	Q2	325 (28.2)	1	Ref.	1	Ref.
	Q3	431 (37.3)	1.39 (1.20–1.61)	<0.001	1.21 (1.04–1.41)	0.016
	Q4	452 (39.2)	1.46 (1.27–1.69)	<0.001	1.21 (1.03–1.42)	0.020
1-year	Q1	458 (39.7)	1.19 (1.05–1.37)	0.009	1.16 (1–1.33)	0.046
	Q2	410 (35.6)	1	Ref.	1	Ref.
	Q3	526 (45.6)	1.37 (1.21–1.56)	<0.001	1.22 (1.06–1.39)	0.005
	Q4	560 (48.5)	1.47 (1.3–1.67)	<0.001	1.24 (1.07–1.43)	0.003

Adjusted model: adjusted for age, gender, heart rate, mean blood pressure, respiratory rate, temperature, SPO_2_, APS III, SOFA, myocardial infarction, congestive heart failure, diabetes mellitus, peripheral vascular disease, cerebrovascular disease, chronic pulmonary disease, chronic kidney disease, severe liver disease, malignant cancer, metastatic solid tumor, white blood cell, hemoglobin, platelets, BUN, calcium, creatinine, glucose, sodium, potassium, total bilirubin, CRRT, invasive ventilation, vasopressor. FAR, fibrinogen to albumin ratio; HR, hazard ratio; CI, confidence interval; SPO_2_, percutaneous oxygen saturation; SOFA, sequential organ failure assessment; APS III, acute physiology score III; BUN, blood urea nitrogen; CRRT, continuous renal replacement therapy.

### FAR and extended mortality analysis

The analysis extended to 90-day and 1-year mortality risks indicated higher risks for lower FAR in group Q1 and higher FARs in groups Q3 and Q4 compared to Q2. Unadjusted HRs were 1.32 (1.14–1.53), 1.39 (1.20–1.61), and 1.46 (1.27–1.69) respectively, with all adjusted HRs—1.25 (1.07–1.46), 1.21 (1.04–1.41), and 1.21 (1.03–1.42)—showing statistical significance. A similar trend was observed in the 1-year mortality risk with unadjusted HRs for groups Q1, Q3, and Q4 at 1.19 (1.05–1.37), 1.37 (1.21–1.56), and 1.47 (1.30–1.67) respectively, and adjusted HRs were 1.16 (1.00–1.33), 1.22 (1.06–1.39), and 1.24 (1.07–1.43). Refer to the Supplementary Table S3 for comprehensive insights.

### Subgroup analysis

In evaluating the efficacy of FAR in predicting primary outcomes across a range of patient subgroups—defined by age (<70 and ≥70 years), gender, severe liver disease, malignant cancer, metastatic solid tumors, sepsis severity (SOFA score <4 and ≥4), and the use of CRRT—an adjusted Cox regression model showed consistent trends across most categories. However, a significant interaction effect among CRRT recipients was noted, substantially impacting the 28-day, 90-day, and 1-year mortality risks, with *p*-values of 0.018, 0.014, and 0.003, respectively. The 28-day mortality risk analysis for these subgroups is detailed in Table 3, with the analyses for 90-day and 1-year outcomes available in the Supplementary Table S4.

**Table 3:** Subgroup analysis of the association between FAR and 28-day mortality.

Subgroups		*N*	Adjusted HR (95% CI)				*P* for interaction
			Q1	Q2	Q3	Q4	
Age (years)	< 70	2,935	1.32 (1.06–1.64)	1 (Ref)	1.09 (0.86–1.38)	0.93 (0.73–1.19)	0.058
	≥ 70	1,680	0.91 (0.67–1.23)	1 (Ref)	1.24 (0.96–1.59)	1.32 (1.02–1.71)	
Gender	Female	1864	1.14 (0.87–1.50)	1 (Ref)	1.21 (0.93–1.58)	1.17 (0.90–1.54)	0.372
	Male	2,751	1.18 (0.95–1.48)	1 (Ref)	1.14 (0.91–1.42)	1.06 (0.84–1.34)	
Diabetes mellitus	No	3,350	1.15 (0.95–1.40)	1 (Ref)	1.11 (0.91–1.36)	1.04 (0.85–1.27)	0.099
	Yes	1,265	1.29 (0.87–1.91)	1 (Ref)	1.41 (1.01–2.01)	1.38 (0.96–1.99)	
Severe liver disease	No	3,928	1.23 (0.99–1.52)	1 (Ref)	1.22 (1.01–1.48)	1.15 (0.95–1.40)	0.158
	Yes	687	1.01 (0.75–1.37)	1 (Ref)	0.91 (0.6–1.36)	0.74 (0.39–1.38)	
Malignant cancer	No	3,973	1.13 (0.93–1.36)	1 (Ref)	1.26 (1.05–1.52)	1.03 (0.84–1.25)	0.661
	Yes	642	1.46 (0.94–2.27)	1 (Ref)	0.82 (0.54–1.24)	1.16 (0.78–1.74)	
Metastatic solid tumor	No	4,376	1.15 (0.96–1.37)	1 (Ref)	1.22 (1.02–1.46)	1.06 (0.88–1.28)	0.560
	Yes	239	1.57 (0.77–3.19)	1 (Ref)	0.72 (0.39–1.32)	1.00 (0.55–1.79)	
SOFA	< 4	638	1.19 (0.54–2.62)	1 (Ref)	1.65 (0.87–3.15)	1.74 (0.91–3.34)	0.125
	≥ 4	3,977	1.19 (1.00–1.42)	1 (Ref)	1.15 (0.97–1.38)	1.06 (0.88–1.27)	
CRRT	No	3,862	1.25 (1.02–1.54)	1 (Ref)	1.38 (1.13–1.68)	1.20 (0.97–1.48)	0.018*
	Yes	753	0.95 (0.70–1.31)	1 (Ref)	0.71 (0.50–1.01)	0.78 (0.55–1.11)	

FAR, fibrinogen to albumin ratio; HR, hazard ratio; CI, confidence interval; SOFA, sequential organ failure assessment; CRRT, continuous renal replacement therapy.

## Discussion

Our findings demonstrated a nonlinear association between admission FAR and prognosis in critically ill sepsis patients, notably affecting both intermediate and long-term prognoses. This outcome underlines the intricate connection between FAR and sepsis prognosis, accentuating its complexity and crucial role in clinical evaluations. Moreover, it offers a potential approach or viewpoint for enhancing the prognostic accuracy of sepsis patients in the ED setting.

Sepsis represents a primary contributor to global mortality and critical illness (24, 25). Inflammation and coagulation function synergistically to protect the host against infection; however, both processes also contribute to tissue damage during the initial phase of sepsis (26, 27). Fibrinogen plays a dual role in sepsis-related inflammation and coagulation. Hyperfibrinogenemia in septic patients stems from elevated fibrinogen production (28) and is closely linked with the severity of sepsis (28, 29). Initially, fibrinogen levels rise as part of the inflammatory response, but levels may decrease in cases like septic shock, indicating worsening conditions (21, 30–34). Hypoalbuminemia in sepsis, reflecting more than just nutritional deficits, is associated with capillary leakage, fluid shifts, and significantly higher mortality and prolonged hospital stays (35–39). Recent research has increasingly utilized albumin as a denominator in prognostic indices for sepsis, such as lactate-to-albumin ratio, C-reactive protein-to-albumin ratio, and procalcitonin-to-albumin ratio, gaining attention for sepsis prognosis in emergency settings (40–42), highlighting its role as a negative acute-phase reactant and a marker of nutritional status. Expanding on this framework, our study investigated the fibrinogen-to-albumin ratio, with fibrinogen—a acute-phase protein integral to coagulation and inflammation—as the numerator. This composite index potentially provides a more comprehensive reflection of the concurrent processes of inflammation and nutritional decline in sepsis.

Our study identified a nonlinear, U-shaped correlation between FAR and the risk of in-hospital mortality in sepsis patients at multiple time points, including 28 days, 90 days, and 1 year. Multifactor-adjusted Cox regression analyses revealed a significantly higher 28-day mortality risk in patients within the first quartile of FAR compared to those in the second quartile. The third and fourth quartiles exhibited an elevated risk, although this was not statistically significant. This pattern contrasts with the positive correlations reported in previous studies. Notably, elevated FAR correlated with increased mortality risk in studies focusing on peritonitis-induced and various infection sites sepsis cohorts (18, 19). We hypothesize that the variability in stages of sepsis and the individual heterogeneity among patients primarily contribute to these differing outcomes. Recent updates to sepsis guidelines have emphasized the importance of long-term patient care, reflecting a growing recognition of the need to focus on long-term outcomes (2). Including out-of-hospital mortality data starting with MIMIC-IV version 2.0 has enhanced our understanding of long-term prognosis. Our findings indicated a consistent nonlinear relationship between FAR and mortality at 90 days and 1 year, with the first, third, and fourth quartile groups displaying significantly higher mortality hazards than the second.

Our findings suggest a more intricate interaction between fibrinogen and albumin levels in sepsis. FAR may reflect a balance of physiological states influenced by the acute phase response, nutritional status, and coagulation factors. While a linear association between albumin levels and mortality risk is well-established, the intricate interplay between fibrinogen levels and mortality risk adds a layer of complexity. However, a prior MIMIC database study revealed a linear, inverse relationship between fibrinogen levels and mortality risk in sepsis patients (43). Additionally, another multicenter cohort study reported that sepsis patients with reduced fibrinogen levels experienced worse outcomes, demonstrating a J-shaped nonlinear correlation with markedly higher death risk at deficient fibrinogen levels (44). The nonlinear, U-shaped relationship between the FAR and mortality likely reflects the complex interplay between its constituent components. Elevated FAR values, often resulting from increased fibrinogen and/or decreased albumin, may indicate pronounced inflammation, hypercoagulability, or malnutrition, all of which contribute to poor clinical outcomes (45–47). Conversely, very low FAR values may also be associated with heightened mortality risk, potentially signaling inadequate fibrinogen levels due to severe hepatic dysfunction, disseminated intravascular coagulation, or immune exhaustion (31, 48, 49). In such cases, the inability to mount an appropriate fibrinogen-mediated inflammatory or coagulative response may indicate advanced disease or organ failure, particularly in late-stage sepsis. Thus, both extremes of the FAR spectrum may reflect physiologically compromised states, accounting for the observed U-shaped risk pattern.

Subgroup analyses revealed no significant interactions in age, gender, diabetes, severe liver disease, malignancy, metastatic solid tumor, or sepsis severity (SOFA score), except for a notable interaction in CRRT patients. CRRT significantly influences fluid and electrolyte balance, potentially affecting protein distribution and levels. The intricate relationship between CRRT and protein levels, such as fibrinogen, is particularly significant. It has been suggested that CRRT can lead to a prothrombotic state, altering fibrinogen levels in a manner akin to nephrotic syndrome (50). The role of fibrinogen in acute ischemic kidney injury was also investigated, revealing that its complete absence is harmful, but partial reduction can improve outcomes (51). In this patient subgroup, we observed that the hazard ratio for mortality in the third quartile group of FAR appeared to be lower than in the other quartile groups. However, this difference did not reach statistical significance. This observation, diverging from the general trend, hints at a possible nonlinear relationship between FAR and mortality risk. Acknowledging these subtleties is vital for precise risk assessment. Further research is necessary to investigate the mechanisms behind these trends and ascertain if these findings are consistent in larger cohorts or different clinical contexts.

To our knowledge, this study represents the most extensive retrospective cohort analysis examining the association between admission FAR and mortality in critically ill sepsis patients, marking the first report of a nonlinear relationship between these factors. However, several limitations must be acknowledged: First, the retrospective design may have led to the omission of critical variables due to data insufficiency. Fibrinogen and albumin data were unavailable for approximately two-thirds of sepsis patients meeting the inclusion criteria. Future studies could benefit from a prospective approach to ensure comprehensive data collection, including detailed medication use and precise indicators of acute inflammatory states during blood sampling. Second, our analysis was limited to initial post-admission FAR values without considering variations during hospitalization. Future research should investigate dynamic FAR assessment throughout the hospital stay to potentially reveal a more nuanced understanding of its predictive value. Third, as a single-center study, selection bias could affect our findings. Multicenter studies are recommended to enhance generalizability, reduce biases, and include a diverse patient population, thereby providing broader insights into the prognosis of FAR in sepsis.

## Conclusion

The U-shaped relationship between FAR at admission and both short-and long-term mortality hazard in patients with sepsis underscores the need for careful interpretation of FAR in clinical settings. Both extremely high and low FAR values, with a nadir at 94.5*10^−3^, correlate with poor outcomes, highlighting the importance of nuanced biomarker assessment. These results emphasize the need for more personalized sepsis management strategies. Future research should aim to validate these findings and clarify the mechanisms behind this nonlinear association, considering potential confounding factors.

## Data Availability

Publicly available datasets were analyzed in this study. The datasets used in this study are publicly available: the MIMIC-IV database (https://physionet.org/content/mimiciv/3.0/).

## Footnotes

^1^ http://www.R-project.org
